# Graphenylene Nanoflakes: A Promising Platform for Toxic Gas Detection

**DOI:** 10.1021/acsomega.6c01694

**Published:** 2026-05-23

**Authors:** Gabriel H. Batista, Ricardo Paupitz, Thomas Niehaus

**Affiliations:** † Physics Department, São Paulo State University - UNESP, Rio Claro, São Paulo 13506-900, Brazil; ‡ Institut Lumiere Matière (iLM), UMR 5306, CNRS, 27098 Universite Claude Bernard Lyon 1, Villeurbanne 69622, France

## Abstract

Gas monitoring, especially for toxic gases, is essential for various applications, including environmental pollution detection, industrial process control, and safety systems. In this regard, two-dimensional (2D) materials have attracted significant interest due to their unique electronic and structural properties, which can be tailored to enhance interactions with the target molecules. In this context, graphenylene nanoflakes, a porous 2D material, emerge as promising candidates due to their unique hexagonal network structure and tunable electronic properties. In this study, we use a Density Functional Theory based method to investigate theoretically the adsorption of various analytes, including some toxic gases, on graphenylene nanoflakes functionalized with transition metals (TMs). Our goal is to evaluate the adsorption capabilities of these graphenylene-based systems and how this adsorption affects the optical absorption spectrum, enabling the material to function as a gas sensor. Our results indicate that the adsorption energies of the selected analytes are significantly higher than those observed in TM-doped graphene flakes, suggesting stronger interactions. Furthermore, in certain cases, the adsorptions led to noticeable changes in the optical absorption spectra, paving the way for gas detection based on such effect. These findings can contribute to the development of new graphenylene-based sensing platforms with enhanced sensitivity and selectivity.

## Graphical abstract

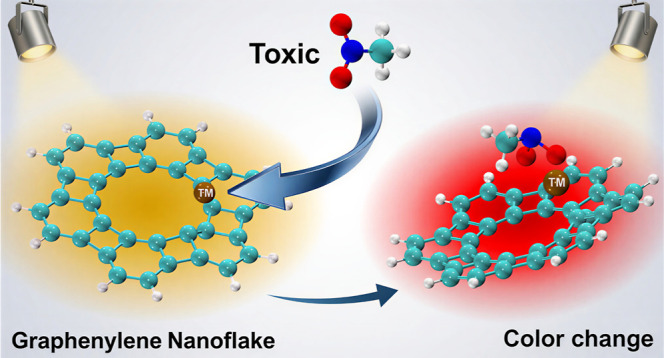

## Introduction

In the context of a complex and technologically diverse society, the exploration of novel materials and the search for new fabrication methods is always relevant. An undesired side effect is the production of hazardous materials that can be produced as a result of industrial chemical processes or as unexpected contamination. In this sense, detecting toxic gases is crucial for instance in environmental monitoring, industrial safety, and public health protection. Development of high-performance gas sensors can be of major importance and the demand for sensitive, selective, and rapid-response gas sensors is increasing.^1–3^

Two-dimensional (2D) materials have garnered substantial attention due to their unique mechanical, electronic and optical properties. This class of material has also been considered of great value due to a diverse set of chemical functionalization options that can alter their physicochemical properties, which can be highly beneficial for gas-sensing platforms.^2,4^ Since the pioneering isolation of graphene, a variety of graphene-like 2D materials - such as transition-metal dichalcogenides, hexagonal boron nitride, graphynes, biphenylene networks and, more recently, graphenylene, have emerged as next-generation candidates for nanoscale sensor applications.^4^

This study focuses on graphenylene, a recently synthesized^5^ porous two-dimensional material which is composed of sp^2^-hybridized carbon atoms arranged in a regular lattice. Its structure has the same architecture as graphene, represented in Figure 1a, however, in this case the repeating unit is composed of biphenylene molecules. This combination results in a 2-dimensional material with periodic four-, six-, and 12-membered units, as illustrated in Figure 1b. This geometry features the symmetry of the *P*6/*mmm* space group and its central dodecagonal nanopore has a diameter of 5.47 Å.^6^ In contrast to established 2D sensing materials such as graphene and molybdenum disulfide (MoS_2_),^7–9^ which predominantly operate via chemiresistive mechanisms, graphenylene offers a promising platform for optical sensing owing to its intrinsic porosity and discrete electronic states. Its pores act as natural anchoring sites for transition metal atoms, effectively preventing their aggregation. In graphene, by contrast, defect engineering is typically required to achieve similar stabilization.^10^

**Figure 1:**
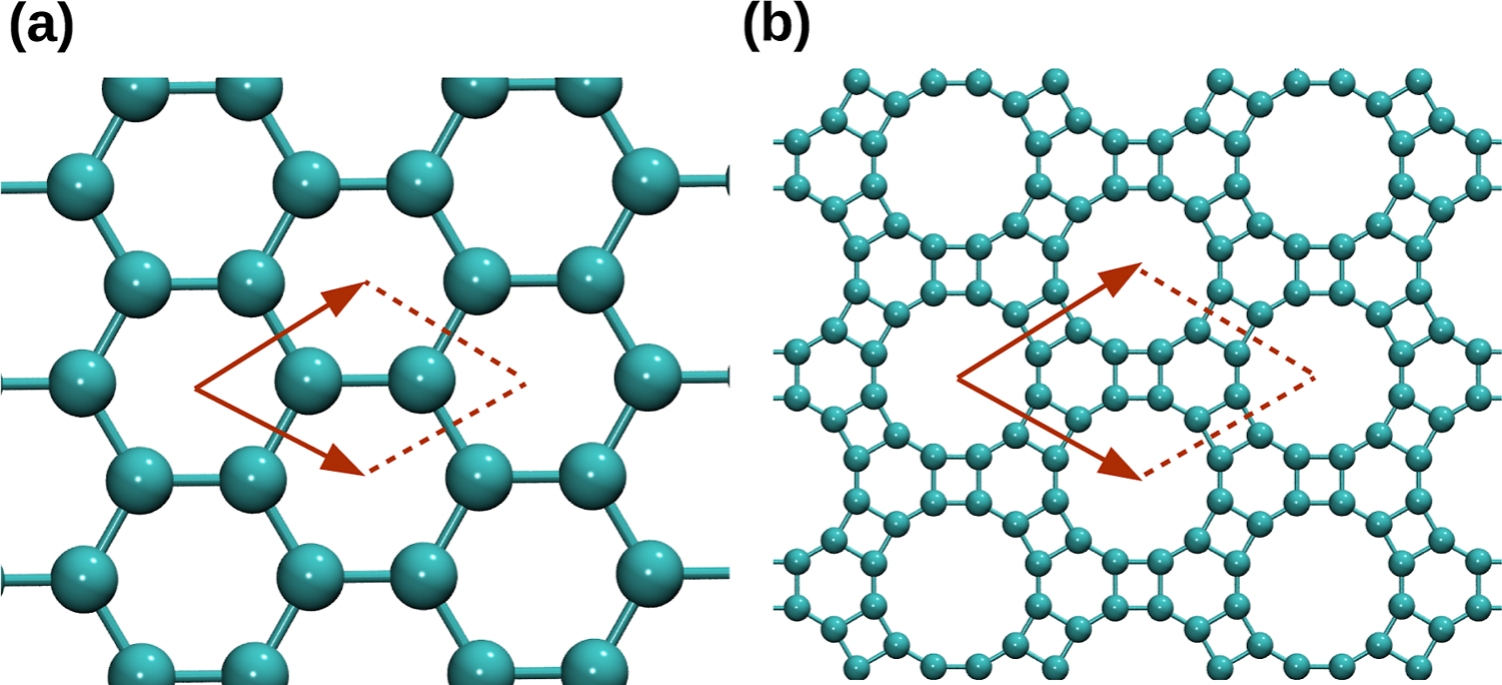
Representation of the unit cells of (a) graphene and (b) graphenylene.

Beyond its unique lattice topology, theoretical and computational studies confirm that graphenylene-based nanoflakes possess robust mechanical stability, tunable electronic band structures, and promising adsorption characteristics when compared to traditional carbon materials and other two-dimensional systems.^11–13^ These characteristics have motivated several applications. In the field of energy storage, graphenylene has been investigated for Li^14^ and Na–ion batteries,^15^ as well as for hydrogen storage.^16^ In catalysis, it has been demonstrated that Ru- and Mo-supported graphenylene can promote CO and NO oxidation,^17^ while the adsorption of transition metals such as Mn, Co, Ni, Pt, and Pd can further enhance its catalytic performance.^18,19^

Considering the influence of transition-metal functionalization on the properties of graphenylene, in the present study we investigate whether these modifications can also enhance its ability to detect certain compounds of interest. For this end, the investigation includes known toxic gases, such as carbon monoxide (CO), carbon dioxide (CO_2_), hydrogen cyanide (HCN), methyl isocyanate (CH_3_NCO), nitrous oxide (N_2_O), nitroethane (C_2_H_5_NO_2_) and nitromethane (CH_3_NO_2_). Two larger molecules which pose significant health or environmental risks were also considered, namely triphenylamine ((C_6_H_5_)_3_N), and lysergic acid diethylamide (C_20_H_25_N_3_O, LSD). CO is a colorless, odorless toxic gas responsible for numerous poisoning incidents worldwide.^20,21^ CO_2_, although less toxic, is a major greenhouse gas with sensors widely studied for environmental monitoring.^22,23^ HCN is a highly toxic gas with industrial relevance and one example of gas whose detection can be achieved using electrochemical sensors.^24^ CH_3_NCO is a toxic compound generated from agricultural activities, industrial sources and biomass burning emissions^25^ for instance. N_2_O is a potent greenhouse gas, mainly emitted from soil management, requiring sensitive sensors for effective environmental monitoring.^26^ Nitroethane and nitromethane are volatile organic compounds with suspected toxicity and use in industrial processes.^27,28^ Triphenylamine is a colorless crystalline organic compound used in functional materials and sensing applications. It exhibits moderate toxicity and may cause irritation to the skin, eyes, and respiratory system upon exposure.^29,30^ LSD, although not a gas, was included as a test molecule for its complex structure and typical challenge in detection applications. These compounds have been widely investigated in gas sensing and broader molecular sensing applications due to their importance in health, safety, and environmental contexts.^31,32^ The selected set provides a broad range of chemical functionalities and toxicities to assess the performance and selectivity of the functionalized graphenylene nanoflakes.

Although not all investigated species are gaseous under typical sensing conditions, for simplicity we hereafter use the term “gases” in a broader sense to refer collectively to all target molecules considered in this work.

Inspired by advances in related two-dimensional systems, such as graphene and hBN, where transition-metal functionalization significantly enhances the adsorption energy of gas molecules,^33,34^ we explore whether similar effects arise in graphenylene. Here, we present a theoretical investigation based on density functional tight-binding (DFTB) calculations to analyze the adsorption of various molecules—including toxic gases—on transition-metal-functionalized graphenylene nanoflakes, with particular emphasis on how these interactions modify their optical absorption spectrum. By correlating adsorption behavior with optical response, our results highlight the potential of graphenylene nanoflakes as effective platforms for gas detection, paving the way for the development of next-generation optical gas sensors with enhanced sensitivity and selectivity.

## Methodology and Computational Details

The Density Functional based Tight Binding (DFTB) method^35^ was employed for geometry optimizations and adsorption energy calculations. DFTB is an approximate quantum mechanical approach derived from density functional theory (DFT),^36,37^ where the total energy is expanded in terms of charge density fluctuations using precomputed integrals. This approximation reduces the computational cost while retaining the essential features of the electronic structure, making it suitable for the simulation of large-scale molecular and material systems.

DFTB preserves most of DFT’s strengths, but some of its limitations are also inherited. One example is the fact that it remains suitable for ground-state properties, but is inaccurate when applied to the description of excited states.^38^ In this context, taking into account that our interest is to study the absorption spectra of several molecular systems, the use of time dependent-DFT­(TD-DFT) methods is necessary. Excited-state properties and optical absorption spectra were evaluated using the linear-response time-dependent DFTB (TD-DFTB).^38,39^ The formalism is based on the approach proposed by Casida for solving the random phase approximation (RPA) equations.^39^ In the linear response regime, perturbation theory can be employed to describe spectroscopic properties. Considering weak fields as the external perturbation on the system, the most important function to be examined is the susceptibility χ which, in the frequency domain can be written as a Dyson-like equation^39^

$$\begin{array}{ll} \chi \left(r,r^{\prime }\right) & =\chi_{KS}\left(r,r^{\prime }\right)\\ & +\int d^{3}r_{1}\int d^{3}r_{2}\chi_{KS}\left(r,r_{1}^{\prime },\omega \right)\left\{\frac{1}{\left|r_{1}-r_{2}\right|}+f_{xc}\left(r_{1},r_{2},\omega \right)\right\}\chi \left(r_{2},r^{\prime },\omega \right), \end{array}$$ 1

where *f*_xc_ is the exchange–correlation kernel and χ_KS_ is the analog of the susceptibility for the ground-state Kohn–Sham system (for more details see the discussion in Chapter 1 of Marques et al.^39^). This susceptibility presents poles for the transition frequencies of the system which are directly related with the optical absorption peaks. In order to determine the poles corresponding to singlet excited states of the system it is necessary to solve the eigenvalue equation

2 $$\sum_{q^{\prime }}R_{qq^{\prime }}F_{q^{\prime }}=\Omega^{2}F_{q}$$

where $R_{qq^{\prime }}=\omega_{q}^{2}\delta_{qq^{\prime }}+4\sqrt{\omega_{q}\omega_{q^{\prime }}}K_{qq^{\prime }}$ , while the coupling matrix *K* is defined as

$$K_{qq^{\prime }}=\int d^{3}r\int d^{3}r^{\prime }\xi_{q}^{*}\left(r\right)f_{Hxc}\left(r,r^{\prime }\right)\xi_{q^{\prime }}\left(r^{\prime }\right)$$ 3

with the Hartree exchange–correlation kernel defined as

$$f_{Hxc}\left(r,r^{\prime }\right)=\frac{1}{\left|r-r^{\prime }\right|}+f_{xc}\left(r,r^{\prime }\right)$$ 4

here the index *q* corresponds to a transition between occupied (*i*) and virtual (*a*) molecular orbitals ψ­(***r***) and ξ_*q*_(***r***) = ψ_*i*_(***r***)­ψ_*a*_(***r***). The application of this approach in the context of DFTB considering the Mulliken approximation, allows the simplification of the coupling matrix *K* and the solution for the excited states energies as well as the eigenvectors *F*, which are used to calculate the oscillator strength for transition *I* ^38^ according to

$$f_{I}=\frac{4}{3}\Omega_{I}\underset{k=x,y,z}{\sum }{\left|\underset{ia}{\sum }\underset{A}{\sum }R_{A,k}q_{A}^{ia}\sqrt{\frac{\omega_{ia}}{\Omega_{I}}}F_{ia}^{I}\right|}^{2}$$ 5

which enable the construction of absorption spectra. Here, ***R***_A,k_ denotes atomic positions, *q*_A_^*ia*^ are transition charges and ω_*ia*_ is the energy difference between orbitals *a* and *i*. Further details of the formalism are provided in refs 38 and 40.

The TD-DFTB approach preserves the essential physics of TD-DFT providing reliable excitation trends and valuable insight into charge redistribution and optical response in nanostructured systems.^41–46^

Beyond the quantitative analysis of absorption spectra, an additional postprocessing step was implemented to translate the calculated optical responses into visually perceivable colors, enabling intuitive interpretation of spectral variations upon gas adsorption. For each optical absorption spectrum obtained in our calculations, we used a homemade python code to carry a standard colorimetric transformation pipeline as described elsewhere^47,48^ in order to obtain an estimated perceivable color for the human eye.

The conversion follows a well-defined sequence of transformations from the absorption domain to the sRGB color space. First, the transmittance spectrum was computed as *T*(λ) = 1 – *A*(λ), where *A*(λ) is the normalized absorbance obtained from TD-DFTB calculations. The transmittance was then weighted by the CIE 1931 ^49^ color matching functions (*x̅*(λ), *y̅*(λ), *z̅*(λ)) and the spectral power distribution of the D65 illuminant^50^ *S*(λ) to yield the CIE tristimulus values

$$X=\int_{\lambda }\mathrm{x̅}\left(\lambda \right)T\left(\lambda \right)S\left(\lambda \right)d\lambda ,$$ 6

$$Y=\int_{\lambda }\mathrm{y̅}\left(\lambda \right)T\left(\lambda \right)S\left(\lambda \right)d\lambda ,$$ 7

$$Z=\int_{\lambda }\mathrm{z̅}\left(\lambda \right)T\left(\lambda \right)S\left(\lambda \right)d\lambda ,$$ 8

where the integration is performed over the visible range (380–780 nm, 1.6–3.1 eV). These values represent the spectral color stimulus in the CIE *XYZ* space. The resulting *XYZ* coordinates were then converted to linear RGB components through standard linear and gamma corrections, enabling visualization of each configuration’s color. This procedure allows direct visual comparison of optical modifications induced by molecular adsorption, providing an intuitive representation of the system’s photoresponse. While this procedure allows for a direct visual comparison of optical modifications, it should be interpreted as a qualitative representation rather than a definitive prediction of physical appearance. This mapping is inherently sensitive to the predictive accuracy of the underlying TD-DFTB calculations, as minor shifts in excitation energies can lead to non-negligible variations in the rendered sRGB hue. Furthermore, this idealized model assumes a standard D65 illuminant and neglects complex experimental variables, such as sample concentration, size dispersion, and scattering effects, that would influence the perceived color of a physical sample in a laboratory setting.

DFTB and TD-DFTB calculations were carried out using the DFTB+ code,^46^ within the self-consistent charge (SCC-DFTB) formalism and a convergence threshold of 1 × 10^–6^ a.u. for Mulliken charge fluctuations. The Slater–Koster parameter sets TRANS3D^51^ and MIO^52^ were employed, considering collinear spin polarization. To determine the number of unpaired electrons in each functionalized system, we tested different total spin values—from 0 to 5 unpaired electrons—since transition metals can exhibit multiple spin states. For each case, the configuration with the lowest total energy was selected. The resulting spin values were then adopted for all molecule-adsorbed systems, as the adsorbates considered are diamagnetic and therefore do not introduce additional unpaired electrons.

Dispersion corrections were included via UFF-based Lennard–Jones terms, and all geometry optimizations and SCF cycles followed standard DFTB+ settings. The number of excited states was chosen to cover the spectral range of 1.6–3.1 eV, typically from a few hundred for small systems to over 600 for the largest ones, ensuring spectral convergence.

The graphenylene nanoflake (GNF) was modeled as the smallest cluster preserving the intrinsic symmetry of graphenylene, namely the hydrogen-passivated hexagonal molecule C_36_H_12_ represented in Figure 2. This small cluster size was chosen in order to maintain a manageable computational cost during our simulations.

**Figure 2:**
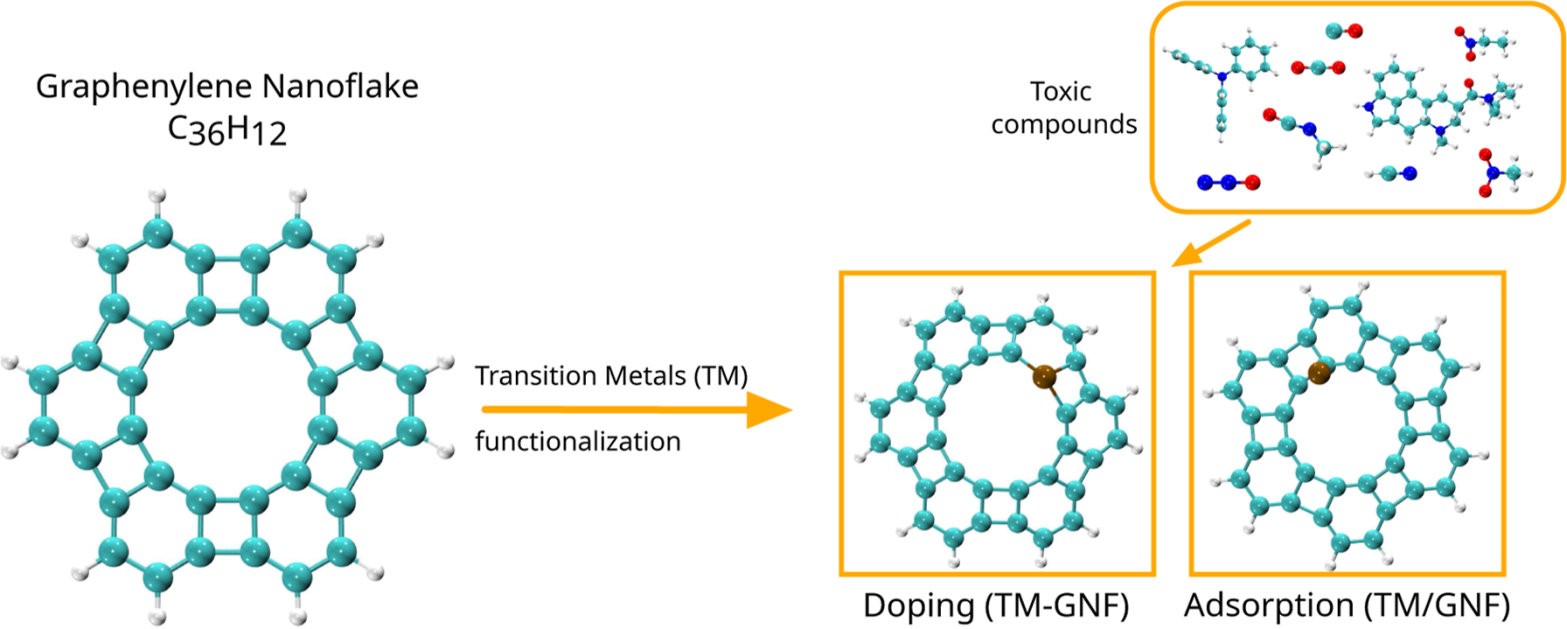
Schematic representation of the graphenylene nanoflake (GNF) and two functionalization schemes: transition metal doping (TM-GNF) and transition metal adsorption (TM/GNF), both followed by gas adsorption. The upper inset illustrates a selection of gases (some of them toxic) studied in this work.

As indicated in Figure 2, we distinguish two different functionalization schemes: substitutional doping (TM-GNF), in which one carbon atom is replaced by a transition-metal atom (Co, Fe, Ni, Sc, or Ti), and metal adsorption (TM/GNF), in which the metal atom is adsorbed on the nanoflake surface without lattice substitution. For the doping case, different substitutional sites were considered. For the adsorption case, the metal atom was initially placed about 3 Å above different trial sites (center, hexagonal ring, square ring, and edge), followed by full structural relaxation. The lowest-energy configuration was then selected in each case.

A set of toxic and environmentally relevant compounds was adsorbed onto these functionalized graphenylene nanoflakes to evaluate their sensing potential. The selected compounds—CO, CO_2_, HCN, CH_3_NCO, N_2_O, C_2_H_5_NO_2_, CH_3_NO_2_, triphenylamine ((C_6_H_5_)_3_N), and LSD (C_20_H_25_N_3_O)—span a wide range of chemical functionalities and toxicities. This selection provides a broad testing ground for evaluating the performance and selectivity of the TM-functionalized graphenylene nanoflakes.

To validate the DFTB parametrization, complementary DFT calculations were carried out on small a graphene nanoflake—coronene—using the NWChem package.^53^ The comparison shows that DFTB tends to overestimate the adsorption energy compared to DFT, with deviations typically ranging from ≈0.3 to 0.7 eV. For the Ni–O case, a deviation from this trend was observed, consistent with the known limitations of standard DFTB parametrizations for such interactions.^54^ Computational details and validation results are provided in Section S1 of the Supporting Information.

The adsorption energies (*E*_ads_) of gas molecules on pristine, doped­(TM–GNF), and adsorbed­(TM/GNF) graphenylene nanoflakes were calculated using eqs 9–11

9 $$E_{ads}=E_{gas/GNF}-E_{GNF}-E_{gas},$$

10 $$E_{ads}=E_{gas/TM-GNF}-E_{TM-GNF}-E_{gas},$$

11 $$E_{ads}=E_{gas/TM/GNF}-E_{TM/GNF}-E_{gas}.$$

Here, *E*_gas/GNF_, *E*_gas/TM–GNF_, and *E*_gas/TM/GNF_ represent the total energies of the gas molecule adsorbed on the pristine, TM–GNF, and TM/GNF systems, respectively. *E*_GNF_, *E*_TM–GNF_, *E*_TM/GNF_, and *E*_gas_ correspond to the total energies of the pristine GNF, TM–GNF complex, TM/GNF complex, and isolated gas molecule. Negative *E*_ads_ values indicate energetically favorable adsorption processes.

## Results and Discussion

Figure 3 shows optimized structures of GNFs doped with Co, Fe, Ni, Sc, and Ti (first row) and of GNFs adsorbed with Co/, Fe/, Ni/, Sc/, and Ti/ (second row). In both cases, functionalization induces out-of-plane distortions on the flake. Upon doping, the replacement of a carbon atom with a transition metal, which has a larger covalent radius, elongates C–TM bonds and breaks the local sp2 symmetry, driving structural rearrangements to minimize the total energy. For adsorption, the distortion may arise from noncovalent interactions or from more localized effects, depending on the position and coordination environment of the transition metal on the flake.

**Figure 3:**
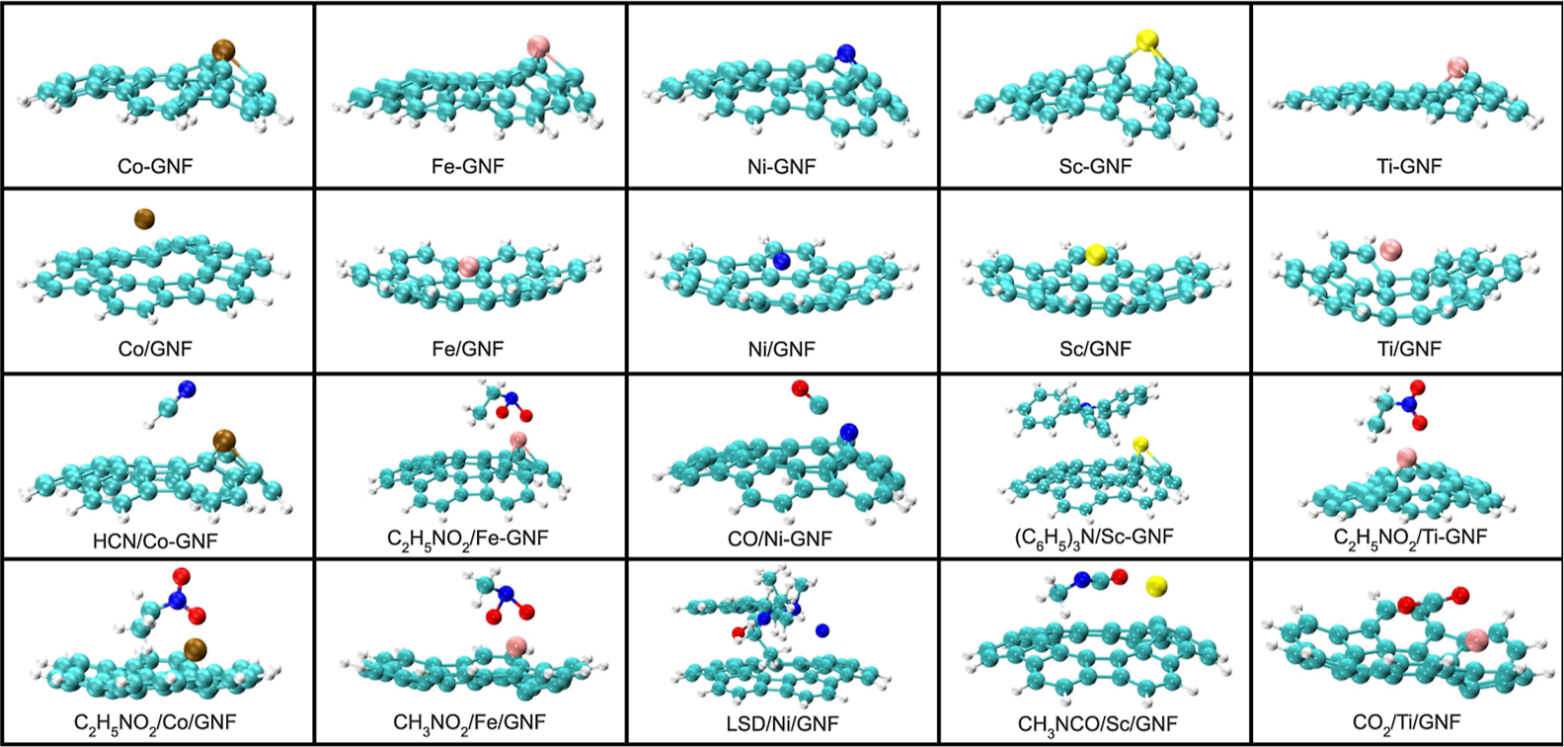
Optimized geometries of functionalized GNFs: substitutional doping with Co, Fe, Ni, Sc, and Ti (row 1); Co/, Fe/, Ni/, Sc/, and Ti/adatoms (row 2); and representative lowest-energy configurations for some complexes (rows 3–4). Carbon in cyan, hydrogen in white, metals and molecular atoms highlighted by color.

Rows 3 and 4 of Figure 3 compile lowest-energy geometries for representative complexes. The complete set is provided in Figure S2 of the Supporting Information. In most cases, molecular adsorption adds small extra distortions and changes some bond lengths and coordination angles around the metal center. In some complexes—such as LSD/Ni/GNF, CH_3_NCO/Sc/GNF, and CH_3_NO_2_/Fe/GNF—the adsorbed molecule drives a relocation of the TM on the GNF, forming a more stable TM-molecule complex that the initial anchoring site on the flake.

Figure 4 shows *E*_ads_ for various molecules on GNF under different functionalization modes, as described above. In general, functionalization significantly increases the absolute value of the adsorption energies for most of the molecules analyzed, compared to pristine GNF. This indicates a greater affinity of these modified surfaces for the adsorbed species. Among the metals studied, titanium stands out consistently, especially in the Ti/GNF arrangement, exhibiting the highest |*E*_ads_| values for most cases. This highlights its potential for applications requiring strong chemical interaction, such as sensors, selective chemical capture, and heterogeneous catalysis.^55,56^

**Figure 4:**
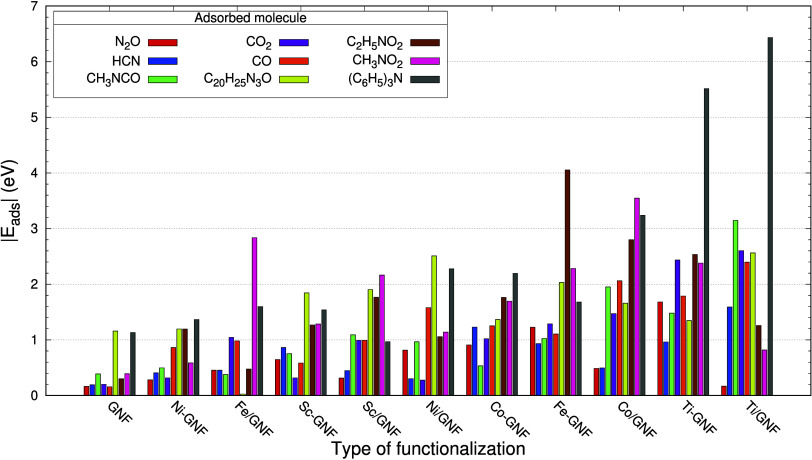
Absolute values of adsorption energies (|*E*_ads_|) for different gas molecules adsorbed on pristine and transition-metal-modified graphenylene nanoflakes. Bars are grouped by GNF functionalization type, while the values correspond to the lowest-energy configuration for each case. Numerical values of adsorption energies are provided in Table S2 of the Supporting Information.

Large organic molecules such as C_20_H_25_N_3_O and (C_6_H_5_)_3_N showed particularly favorable adsorption on Ti- and Ni-decorated surfaces. For example, C_20_H_25_N_3_O/Ti/GNF has *E*_ads_ = −2.5633 eV, for C_20_H_25_N_3_O/Ni/GNF the value is −2.5095 eV, and for (C_6_H_5_)_3_N/Ti/GNF *E*_ads_ = −6.4320 eV. In the latter case, the extremely high value can be attributed to the increased contact surface area and the possibility of multiple π-metal interactions between the molecule and the TM.

When comparing different GNF modification strategies, it is observed that, for several molecules, the Metal/GNF configuration outperforms the metal-GNF in terms of interaction strength. For example, CH_3_NO_2_/Co-GNF: −1.6968 eV > CH_3_NO_2_/Co/GNF: −3.5467 eV, and CH_3_NCO/Ti-GNF: −1.4801 eV > CH_3_NCO/Ti/GNF: −3.1474 eV. These cases suggest that the simultaneous presence of the molecule and a metal as a decorated species on pristine GNF can produce stronger chemical interactions and can promote electronic redistribution.

The results shown in Figure 4 and table S2 suggest titanium—particularly in the Ti/GNF configuration—as the most promising transition metal for applications that require strong molecule–surface interactions. Such strong adsorption, however, may be less suitable for other applications, such as reusable sensors, where a balance between binding strength and ease of desorption is necessary. In cases where chemisorption energies exceed several eV, overcoming the energy barrier typically requires external stimuli, such as thermal annealing or photoregeneration, to recover the active surface. Conversely, these high affinities are advantageous for “one-off” or dosimetric sensors, particularly in toxic gas detection where irreversible binding ensures the permanent capture of hazardous species and a stable signal. In this respect, our results indicate the possibility of chemical selectivity controlled by the choice of dopant.

Likewise, recent studies on Fe-decorated h-BN and Ti-/N–Ti-doped graphene reported enhanced adsorption after transition-metal functionalization, with strong hybridization effects in the former case and stable chemisorption of SO_2_ in the latter, supporting our conclusion that transition-metal functionalization substantially strengthens molecule–surface interactions, particularly for Ti-based systems.^33,56^

Time-dependent DFTB (TD-DFTB) calculations were performed to obtain the optical absorption spectra of the different molecule–GNF complexes functionalized with transition metals. The enhancement in the chemical reactivity of GNFs after TM functionalization leads to noticeable modifications in their optical absorption spectra, as shown in panels (a) of Figure 5I and II.

**Figure 5:**
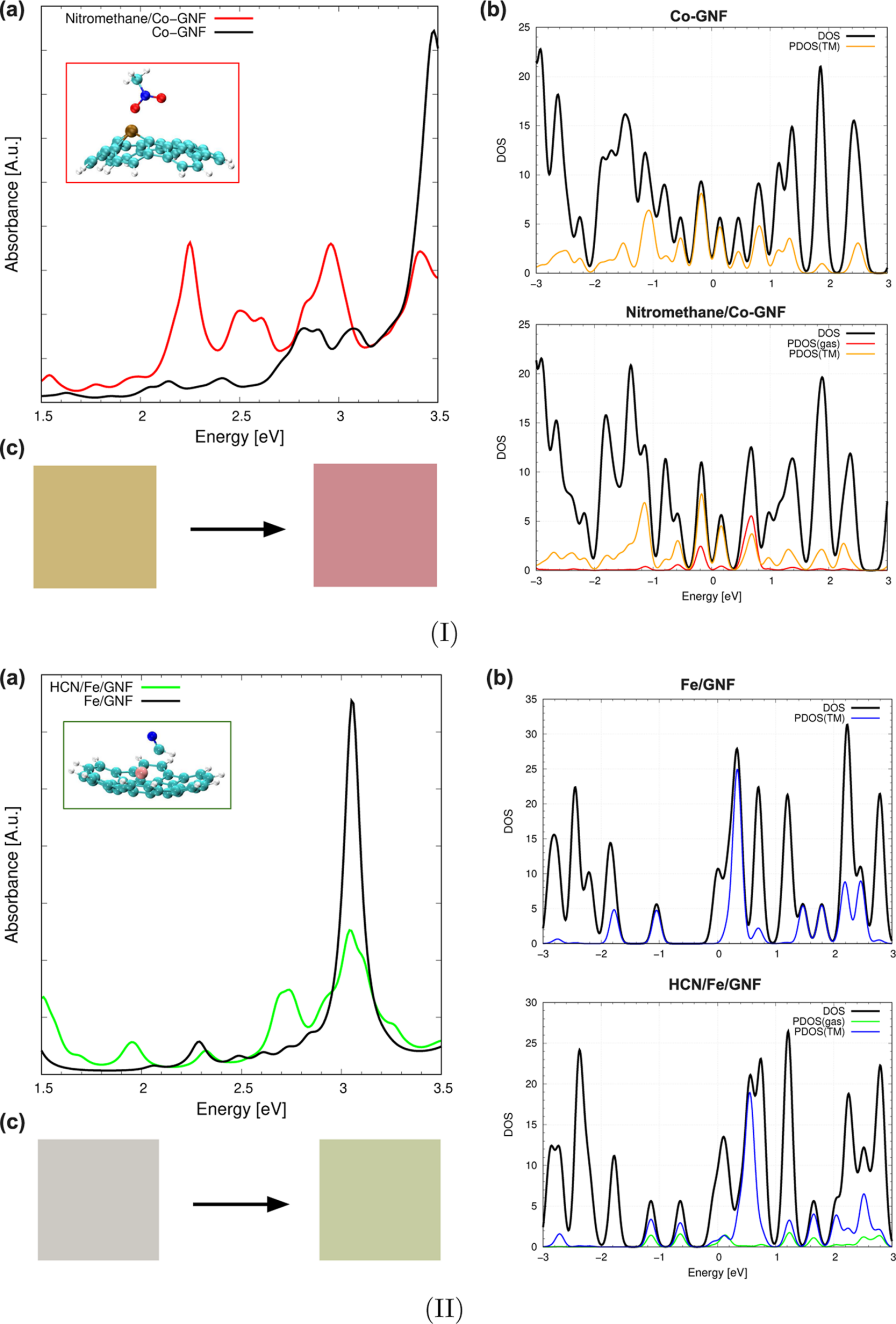
Changes in optical and electronic properties due to gas adsorption in graphenylene nanoflakes functionalized with transition metals. (a) Absorption spectra for Co-GNF (I) and Fe/GNF (II) before (black) and after (red/green) molecule adsorption, revealing clear spectral shifts. Insets show the optimized configurations. (b) Total and projected DOS illustrating adsorption-induced modifications in the electronic structure. (c) Resulting color changes as predicted by the application of the protocol explained in the Methodology Section, evidencing the potential for optical gas detection.

These spectral changes arise from the strong contribution of molecular orbitals associated with the adsorbed gases to the states near the Fermi level (normalized at 0 eV), as shown in the density of states (DOS) plots in panels (b) of the same figures. In general, transitions involving electronic states close to the Fermi level tend to dominate the optical response, so even small perturbations in this region can lead to significant spectral shifts and intensity variations. In particular, the projected DOS (PDOS) indicates hybridization between the TM *d* orbitals and the molecular states, which modifies the energies and symmetries of the frontier orbitals. Consequently, we observe a substantial reorganization of the excited states, with new excitations exhibiting different oscillator strengths and energies, leading to the spectral shifts and intensity redistribution seen in the calculated absorption spectra in panels (a).

Such adsorption-induced variations in the absorption spectrum can manifest as visible color changes. Based on the color perception calculations described in the Methodology section, some systems exhibit clear color shifts upon gas adsorption. In Figure 5I, for instance, the Co–GNF complex shows a golden-like hue before adsorption, which turns reddish after nitromethane adsorption. This qualitative color change is consistent with the redshift and intensity enhancement observed in the absorption spectrum. Similarly, for Fe/GNF in Figure 5II, the color changes from beige/salmon to greenish upon HCN adsorption, reflecting the modification of its electronic transitions.

Other TM/GNF systems also display adsorption-dependent color variations, as illustrated in Figure 6. For Ti/GNF (a), adsorption of HCN, LSD, and nitromethane produces distinct spectral responses, with attenuation and shifts of several peaks. Notably, the attenuation of the black curve peak by approximately 1.6 eV in the other complexes causes the loss of the reddish color of Ti/GNF without adsorption. In Fe/GNF (b), the presence of (C_6_H_5_)_3_N, LSD, or C_2_H_5_NO_2_ molecules similarly alters the optical intensity and peak positions, resulting in a greenish character. Further comparisons for other TM/molecule combinations are provided in Section S4 of the Supporting Information.

**Figure 6:**
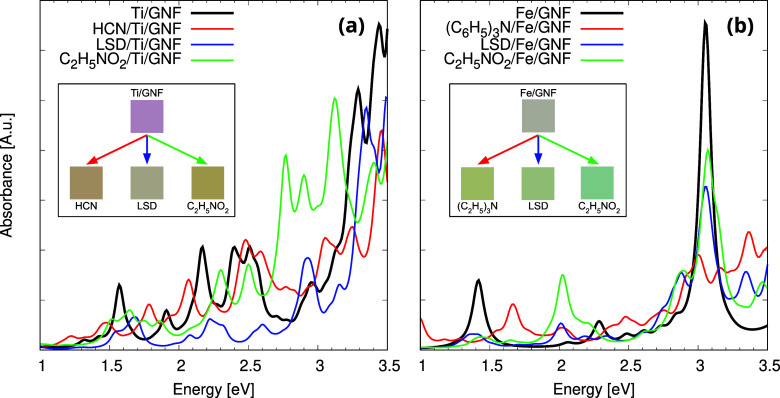
Optical absorption spectra of Ti/GNF and Fe/GNF before and after adsorption of selected molecules in the UV–vis range. Black denotes the TM/GNF, while red/green/blue correspond to the adsorbates listed in each panel, and insets summarize the qualitative color response. (a) Ti/GNF with HCN (red), LSD (green), and nitromethane C_2_H_5_NO_2_ (blue); and (b) Fe/GNF with (C_6_H_5_)_3_N (red), LSD (green), and nitromethane C_2_H_5_NO_2_ (blue). Both show adsorption-induced shifts in peak energies and intensities.

These results indicate that TM-functionalized GNFs may exhibit characteristic optical fingerprints that vary with the nature of the adsorbed molecule. The interaction between the transition metal and the adsorbates modifies the local electronic environment, leading to measurable changes in the visible absorption spectrum. Although these findings are theoretical, they suggest that adsorption-induced optical responses could, in principle, be used for gas detection, where even small shifts in the spectra or color might signal the presence of specific molecules.

To evaluate whether the adsorption-induced optical responses persist at larger scales, we performed tests on a substantially bigger flake (GNF2, C_144_H_24_) functionalized with Ti (see Section S5 of the Supporting Information). Although gas adsorption still modifies the density of states near the Fermi level, the corresponding changes in the absorption spectrum become much less pronounced, likely due to the significantly higher density of states in GNF2. Even multiple adsorption sites (7 Ti + 7 molecules) resulted in only minor spectral variations. This indicates that the pronounced color changes observed throughout this work are rooted in the discrete low-energy excited states characteristic of the TM-GNF combinations studied here, which amplify the optical response to local interactions.

To provide a clearer overview of the main results, a summary is presented below (Table 1). It highlights the general adsorption strength, optical response, possible applications, and main limitations of each TM-functionalized GNF system discussed in this work.

**Table 1:** Condensed Information Regarding Each System Discussed in the Text Considering Adsorption Strength, Optical Response, Plausible Applications and Possible Limitations for Practical Use

system	adsorption strength	optical response	possible applications	limitation
Co-GNF, Co/GNF	moderate to high; Co/GNF can be much stronger than Co-GNF in some cases (e.g., CH_3_NO_2_).	clear spectral and color changes in representative cases.	selective sensing and optical detection.	less consistent than Ti-based systems.
Fe-GNF, Fe/GNF	moderate to high, depending on the adsorbate.	pronounced color and spectral changes in representative cases.	selective sensing and optical detection.	adsorption strength weaker than Ti/GNF in general.
Ni-GNF, Ni/GNF	high for some systems, especially larger molecules.	weak color response.	adsorption for larger molecules.	weak response in the representative cases.
Sc-GNF, Sc/GNF	intermediate, case-dependent.	weak spectral changes and subtle color change in CH_3_NO_2_/Sc-GNF.	case-specific sensing and potentially reversible detection.	weak spectral changes; metal relocation may occur in some complexes.
Ti-GNF, Ti/GNF	highest overall; Ti/GNF gives the strongest adsorption for most molecules.	strong spectral changes in representative cases.	selective chemical capture, dosimetric sensing.	very strong binding may hinder reversibility in reusable sensors.

## Conclusions

In this work, we carried out a theoretical investigation of gas adsorption on transition-metal-functionalized graphenylene nanoflakes using DFTB and TD-DFTB approaches. Our results reveal that functionalization with transition metals significantly enhances the adsorption energy compared to pristine graphenylene, indicating higher surface reactivity and improved affinity toward various gas molecules.

Among the studied metals, titanium stands out as the most promising functionalization, especially in the Ti/GNF configuration, which exhibited the strongest adsorption energies for most adsorbates. This identifies Ti-based systems as particularly attractive for applications requiring strong adsorption, selective chemical capture, and dosimetric sensing, although such strong binding may be less favorable for reusable sensors.

The results also indicate that the sensing performance depends on both the selected metal and the type of molecule adsorbed, revealing a clear possibility of chemical selectivity controlled by dopant choice. In addition, for several molecules, the adsorbed-metal configuration was found to yield stronger interactions than substitutional doping, showing that the functionalization strategy itself is an important parameter for tuning adsorption behavior.

From the optical point of view, some systems exhibited especially clear and potentially useful color changes upon adsorption. In particular, Co-GNF changes from a golden-like hue to a reddish color after nitromethane adsorption, while Fe/GNF changes from beige-salmon to greenish upon HCN adsorption.

These optical effects should be observable in experiments on small nanoflakes, and such flakes could potentially be assembled into thin films for nanoscale optical sensing. Detecting the effects in larger systems may be more challenging. In any case, we hope that the present work provides a useful basis for higher-level theoretical studies and for planning experiments on graphenylene-based gas sensors.

## Supplementary Material



## Notes

DFT validation for DFTB’s predictions, geometries of the full set of optimized structures, adsorption energies, additional examples of adsorption-induced color changes and finite size effects (PDF)
