# Supplementary material for: Graphenylene Nanoflakes: A Promising Platform for Toxic Gas Detection
Source: ACS Omega. 2026 May 23;11(22):32741–50. doi: 10.1021/acsomega.6c01694 (PMC13261593; doi:10.1021/acsomega.6c01694)
Supplement: Supplementary file 1 [file ao6c01694_si_001.pdf]

Supporting Information for: "Graphenylene nanoflakes: A promising platform for toxic gas detection"

Gabriel H. Batista,[*,†] Ricardo Paupitz,[*,†] and Thomas Niehaus[*,‡]

†*Physics Department, São Paulo State University - UNESP CEP-13506-900 Rio Claro, SP, Brazil*

‡*Universite Claude Bernard Lyon 1, Institut Lumiere Matière (iLM), UMR 5306, CNRS, 69622 Villeurbanne, France*

E-mail: gabriel.batista@unesp.br; ricardo.paupitz@unesp.br; thomas.niehaus@univ-lyon1.fr

Contents

S1 DFT validation **2**

S2 Geometries **5**

S3 Adsorption energy values **7**

S4 Other cases of color changing **8**

S5 Finite size effects **11**

S1 DFT validation

In order to validate the results obtained under the approximate method, we performed a direct comparison between DFTB's predictions and DFT calculations for representative systems. DFT calculations were carried out using the NWChem software[1] with the PBE functional[2,3] and def2-SVP basis set.[4] Default tight convergence criteria were applied, ensuring accuracy for both self-consistent field (SCF) and geometry optimizations. For SCF, convergence was achieved when the energy change was below 1×10^{-6} Hartree, the RMS density matrix difference below 1×10^{-5}, and the maximum DIIS error under 5×10^{-4}. Geometry optimization was considered converged when GMAX $< 2.0\times10^{-3}$ a.u., GRMS $< 1.0\times10^{-3}$ a.u., XMAX $< 2.0 \times 10^{-3}$ a.u., and XRMS $< 1.0 \times 10^{-3}$ a.u.

To perform this validation in a computationally efficient, and yet representative way, we selected the coronene molecule ($C_{24}H_{12}$) as a model system. This graphene-like flake preserves the essential structural features of the 2D materials while containing significantly fewer atoms, allowing faster DFT calculations without compromising the physical relevance of the results. The flake was functionalized by adsorbing the same transition metals (TMs) considered in our main graphenylene-based systems.

The validation focused on the adsorption of a CO molecule on TM-functionalized coronene. Adsorption energies were computed using both DFT and DFTB, with the latter employing the same parameters as in the main study. Since our goal was to quantify the deviation of DFTB adsorption energies relative to DFT, all geometries were first optimized at the DFT level and subsequently used as fixed structures for single-point DFTB calculations.

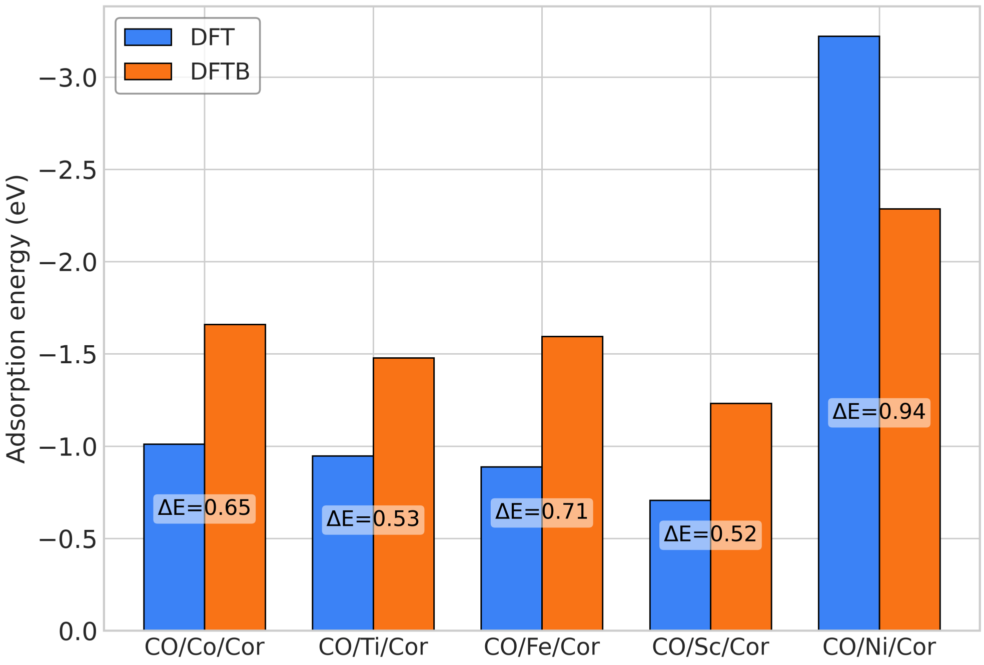

Figure S1: Comparison between DFT and DFTB adsorption energies for CO on transition-metal-functionalized coronene (TM/Cor) systems. Blue and orange bars correspond to DFT and DFTB results, respectively. The values of $\Delta E = E_{ads}(\text{DFTB}) - E_{ads}(\text{DFT})$ represent the deviation between both methods.

Table S1: Numerical comparison between adsorption energies obtained from DFT and DFTB calculations for CO adsorption on transition-metal-functionalized coronene (TM/Cor) systems.

Complex	E_{ads} (DFT) [eV]	E_{ads} (DFTB) [eV]	ΔE[eV]	ΔE[kCal/mol]
CO/Co/Cor	-1.0111	-1.6592	-0.6480	-14.9453
CO/Ti/Cor	-0.9471	-1.4787	-0.5316	-12.2590
CO/Fe/Cor	-0.8881	-1.5948	-0.7067	-16.2970
CO/Sc/Cor	-0.7070	-1.2314	-0.5244	-12.0933
CO/Ni/Cor	-3.2224	-2.2863	0.9361	21.5881

The results presented in Fig. S1 and Table S1 demonstrate consistency and relative accuracy of DFTB in reproducing the adsorption energy trends across different TM/Cor systems. In most cases DFTB overestimates the adsorption energy compared to DFT, with deviations typically ranging from $\approx$ 0.3 to 0.7 eV.

A notable exception is observed for the Ni-containing system, where a significantly larger

deviation appears. This behavior can be explained considering the limitations of standard DFTB parameterizations, particularly those derived from the trans3d Slater–Koster files, when dealing with systems involving transition metals and oxygen-containing adsorbates. As discussed by Hourahine et al.,[5] the self-interaction error and the incomplete treatment of on-site electronic correlations in the d-manifolds may lead to qualitatively incorrect energy levels for strongly correlated materials such as NiO. These inaccuracies arise mainly from the difficulty in correctly parameterizing short-range interactions due to the mixed ionic–covalent and highly localized character of the $3d$ orbitals.

For the graphenylene-based systems investigated in the main text, configurations involving close Ni–O contacts were intentionally avoided. The chosen geometries minimize such problematic interactions, ensuring that DFTB remains within its best accuracy regime and that the obtained adsorption energies reliably reflect the underlying chemical trends.

S2 Geometries

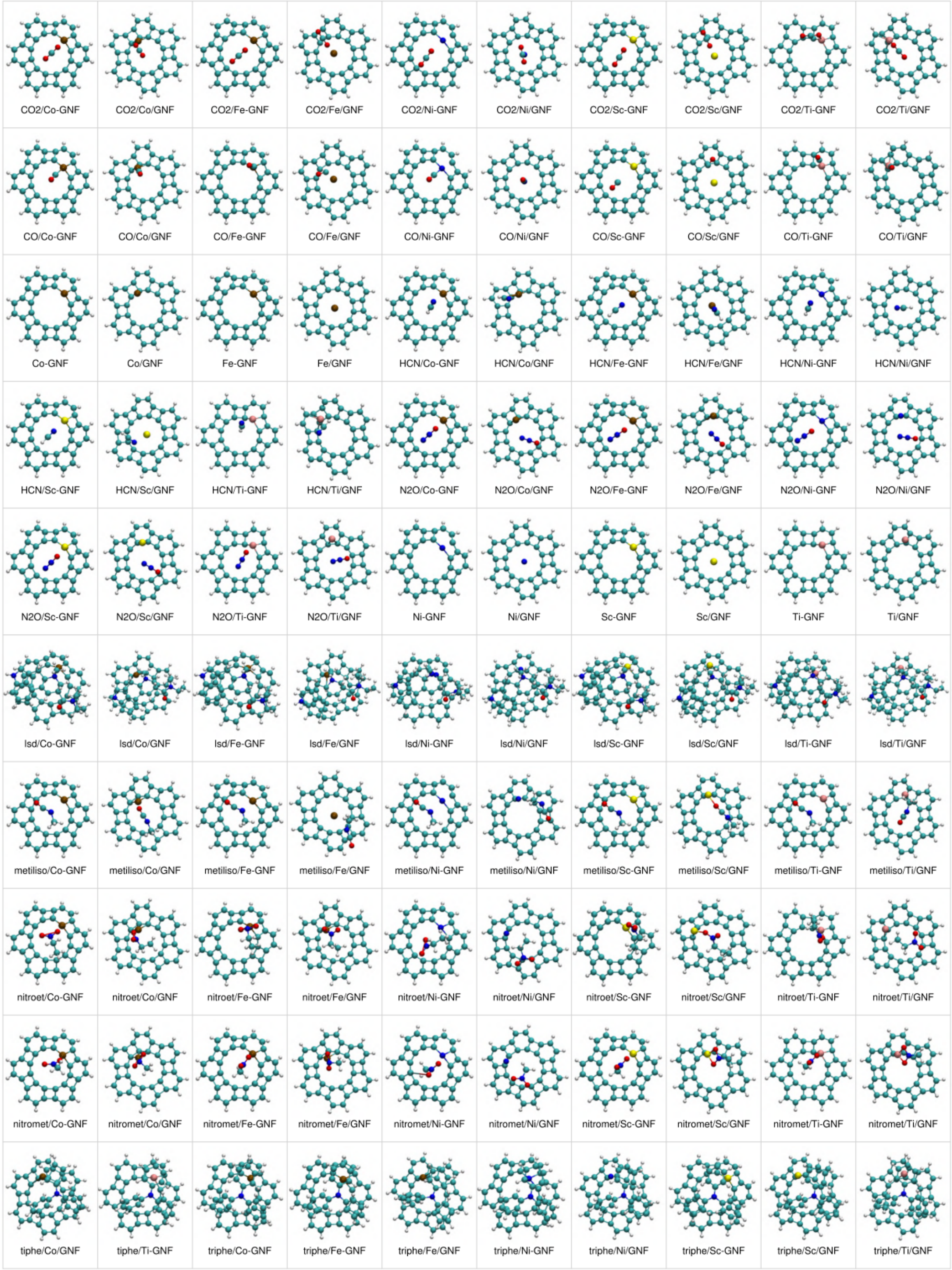

Figure S2: Complete set of optimized geometries for all molecule–TM-GNF complexes investigated in this work. Each panel shows the lowest-energy configuration obtained for a given combination of adsorbate and transition-metal–functionalized GNF (TM = Co, Fe, Ni, Sc, Ti). The collection illustrates the diversity of binding and structural rearrangements induced by different molecules and metals.

Here we present the full set of optimized structures corresponding to all molecule–TM-GNF combinations explored in this study. While Figure 3 of the main text highlights representative cases, the configurations compiled in Fig. S2 provide a comprehensive view of the structural scenario across different transition metals and molecular species. Variations in coordination, adsorption orientation, and local distortion patterns can be observed, reflecting the interplay between the electronic character of the adsorbate and the bonding environment imposed by each transition metal. These geometries form the structural basis for the adsorption energies, and optical response discussed throughout the manuscript.

S3 Adsorption energy values

Table S2: Adsorption energies of selected molecules on GNF and transition metal-functionalized GNF.

Complex	E_{ads} [eV]	Complex	E_{ads} [eV]	Complex	E_{ads} [eV]
CO/GNF	-0.1559	$C_{20}H_{25}N_3O$/GNF	-1.1591	$C_2H_5NO_2$/GNF	-0.3005
CO/Co-GNF	-1.2534	$C_{20}H_{25}N_3O$/Co-GNF	-1.3688	$C_2H_5NO_2$/Co-GNF	-1.7653
CO/Co/GNF	-2.0625	$C_{20}H_{25}N_3O$/Co/GNF	-1.6583	$C_2H_5NO_2$/Co/GNF	-2.7994
CO/Fe-GNF	-1.1056	$C_{20}H_{25}N_3O$/Fe-GNF	-2.0329	$C_2H_5NO_2$/Fe-GNF	-4.0548
CO/Fe/GNF	-0.9828	$C_{20}H_{25}N_3O$/Fe/GNF	-0.0271	$C_2H_5NO_2$/Fe/GNF	-0.4767
CO/Ni-GNF	-0.8623	$C_{20}H_{25}N_3O$/Ni-GNF	-1.1966	$C_2H_5NO_2$/Ni-GNF	-1.1966
CO/Ni/GNF	-1.5789	$C_{20}H_{25}N_3O$/Ni/GNF	-2.5095	$C_2H_5NO_2$/Ni/GNF	-1.0571
CO/Sc-GNF	-0.5852	$C_{20}H_{25}N_3O$/Sc-GNF	-1.8450	$C_2H_5NO_2$/Sc-GNF	-1.2691
CO/Sc/GNF	-0.9928	$C_{20}H_{25}N_3O$/Sc/GNF	-1.9048	$C_2H_5NO_2$/Sc/GNF	-1.7661
CO/Ti-GNF	-1.7887	$C_{20}H_{25}N_3O$/Ti-GNF	-1.3489	$C_2H_5NO_2$/Ti-GNF	-2.5358
CO/Ti/GNF	-2.3970	$C_{20}H_{25}N_3O$/Ti/GNF	-2.5633	$C_2H_5NO_2$/Ti/GNF	-1.2595
CO_2/GNF	-0.1999	CH_3NCO/GNF	-0.3898	N_2O/GNF	-0.1633
CO_2/Co-GNF	-1.0206	CH_3NCO/Co-GNF	-0.5343	N_2O/Co-GNF	-0.9099
CO_2/Co/GNF	-1.4725	CH_3NCO/Co/GNF	-1.9527	N_2O/Co/GNF	-0.4860
CO_2/Fe-GNF	-1.2887	CH_3NCO/Fe-GNF	-1.0265	N_2O/Fe-GNF	-1.2266
CO_2/Fe/GNF	-1.0448	CH_3NCO/Fe/GNF	-0.3774	N_2O/Fe/GNF	-0.4556
CO_2/Ni-GNF	-0.3190	CH_3NCO/Ni-GNF	-0.4979	N_2O/Ni-GNF	-0.2816
CO_2/Ni/GNF	-0.2765	CH_3NCO/Ni/GNF	-0.9653	N_2O/Ni/GNF	-0.8153
CO_2/Sc-GNF	-0.3190	CH_3NCO/Sc-GNF	-0.7513	N_2O/Sc-GNF	-0.6494
CO_2/Sc/GNF	-0.9922	CH_3NCO/Sc/GNF	-1.0910	N_2O/Sc/GNF	-0.3145
CO_2/Ti-GNF	-2.4354	CH_3NCO/Ti-GNF	-1.4801	N_2O/Ti-GNF	-1.6825
CO_2/Ti/GNF	-2.6035	CH_3NCO/Ti/GNF	-3.1474	N_2O/Ti/GNF	-0.1701
HCN/GNF	-0.1931	CH_3NO_2/GNF	-0.3926	$(C_6H_5)_3N$/GNF	-1.1324
HCN/Co-GNF	-1.2294	CH_3NO_2/Co-GNF	-1.6968	$(C_6H_5)_3N$/Co-GNF	-2.1959
HCN/Co/GNF	-0.4950	CH_3NO_2/Co/GNF	-3.5467	$(C_6H_5)_3N$/Co/GNF	-3.2371
HCN/Fe-GNF	-0.9328	CH_3NO_2/Fe-GNF	-2.2821	$(C_6H_5)_3N$/Fe-GNF	-1.6813
HCN/Fe/GNF	-0.4559	CH_3NO_2/Fe/GNF	-2.8361	$(C_6H_5)_3N$/Fe/GNF	-1.5967
HCN/Ni-GNF	-0.4080	CH_3NO_2/Ni-GNF	-0.5865	$(C_6H_5)_3N$/Ni-GNF	-1.3661
HCN/Ni/GNF	-0.3025	CH_3NO_2/Ni/GNF	-1.1399	$(C_6H_5)_3N$/Ni/GNF	-2.2762
HCN/Sc-GNF	-0.8621	CH_3NO_2/Sc-GNF	-1.2856	$(C_6H_5)_3N$/Sc-GNF	-1.5392
HCN/Sc/GNF	-0.4485	CH_3NO_2/Sc/GNF	-2.1664	$(C_6H_5)_3N$/Sc/GNF	-0.9678
HCN/Ti-GNF	-0.9638	CH_3NO_2/Ti-GNF	-2.3807	$(C_6H_5)_3N$/Ti-GNF	-5.5135
HCN/Ti/GNF	-1.5913	CH_3NO_2/Ti/GNF	-0.8203	$(C_6H_5)_3N$/Ti/GNF	-6.4320

S4 Other cases of color changing

Several representative cases of adsorption-induced color changes were discussed in the main text. In this section, we present additional examples in which noticeable variations in the optical response are observed upon gas adsorption. These cases follow the same physical mechanisms described previously, as the modification of the electronic structure near the Fermi level due to hybridization between the transition metal *d* orbitals and molecular states, which leads to changes in the absorption spectrum and, consequently, in the perceived color.

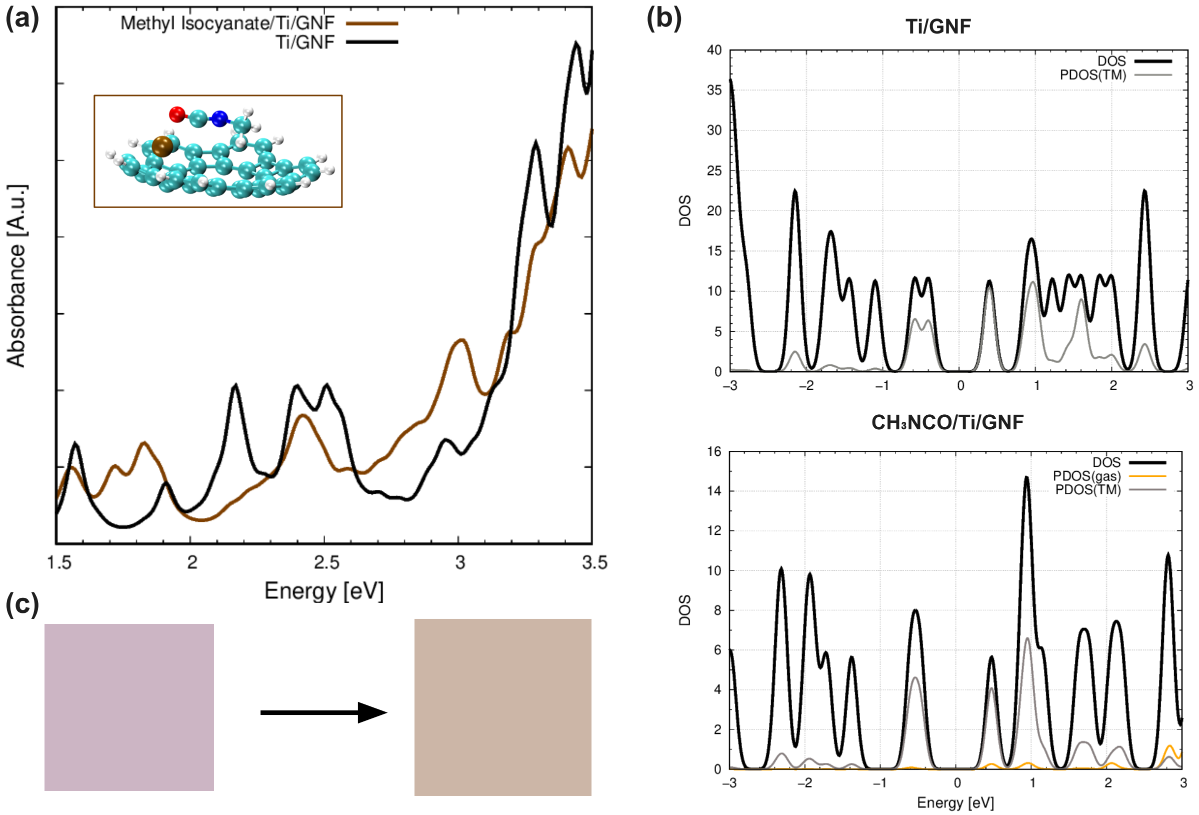

Figure S3: (a) Optical absorption spectra of the Ti/GNF (black) and CH_3NCO/Ti/GNF (brown) systems, highlighting the changes induced by molecule adsorption. The inset shows the optimized structure of Methyl Isocyanate on Ti/GNF. (b) Total and projected DOS (DOS, PDOS) for Ti/GNF (top) and CH_3NCO/Ti/GNF (bottom), emphasizing the electronic modifications upon adsorption. (c) Schematic illustration of the color change associated with the adsorption process.

The corresponding DOS and PDOS plots in Fig. S3(b) reveal that the adsorption of

CH_3NCO induces noticeable changes near the Fermi level, particularly in the higher-energy occupied states. In addition, molecular contributions are observed in these frontier states (yellow curve). Although relatively small, these contributions are sufficient to promote a reorganization of the optical transitions.

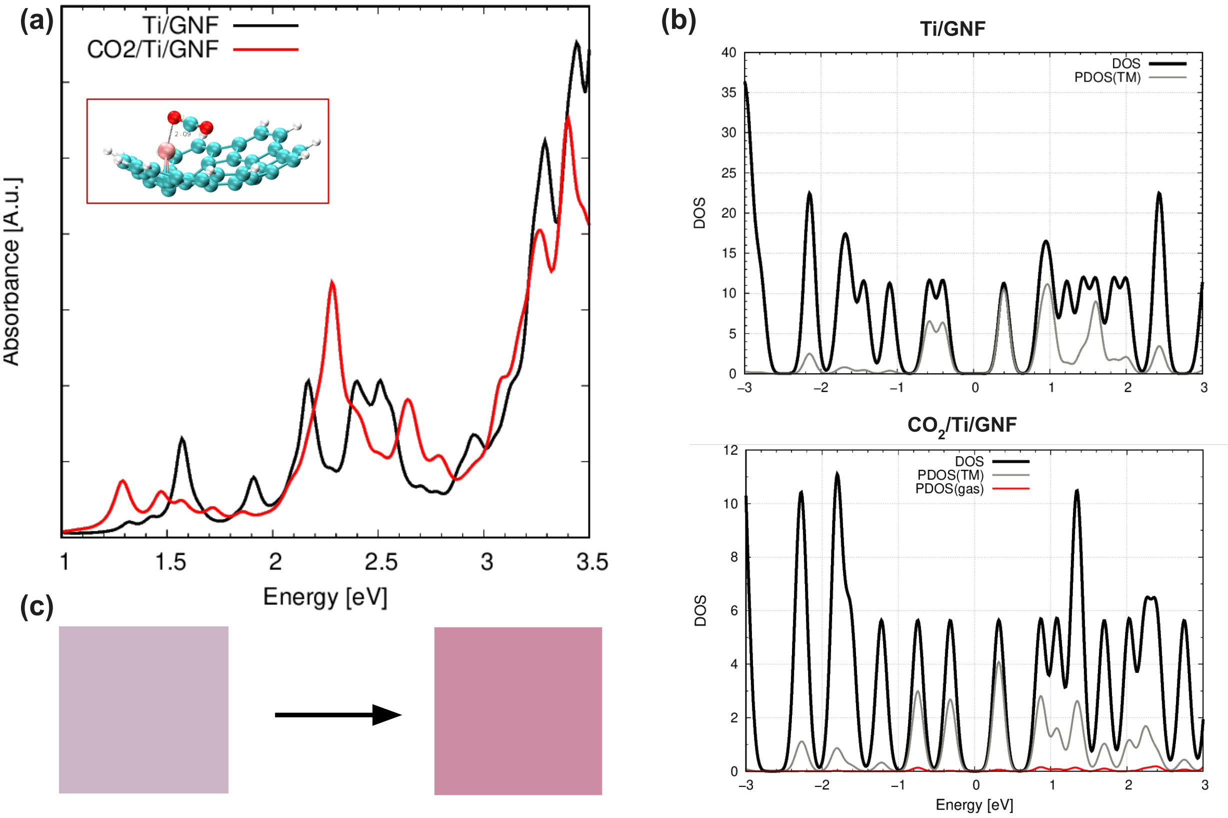

Figure S4: (a) Optical absorption spectra of Ti/GNF (black) and CO_2/Ti/GNF (red), evidencing the spectral variations arising from CO_2 adsorption. The inset displays the optimized configuration of the adsorbed molecule on the Ti/GNF surface. (b) Total and projected DOS (DOS, PDOS) for Ti/GNF (top) and CO_2/Ti/GNF (bottom), showing the changes in the electronic structure introduced by adsorption. (c) Schematic illustration of the color shift associated with the adsorption.

A similar behavior is observed for the adsorption of CO_2 on Ti/GNF, as shown in Fig. S4. Although CO_2 interacts more weakly with the surface compared to CH_3NCO, its adsorption still produces modifications in the absorption spectrum, as displayed in Fig. S4(a). These changes are sufficient to induce a qualitative shift in the resulting color, as illustrated in Fig. S4(c). The DOS analysis in Fig. S4(b) indicates changes in the HOMO-LUMO gap and

frontier orbital distributions, reflecting the coupling between the adsorbed molecule and the Ti center.

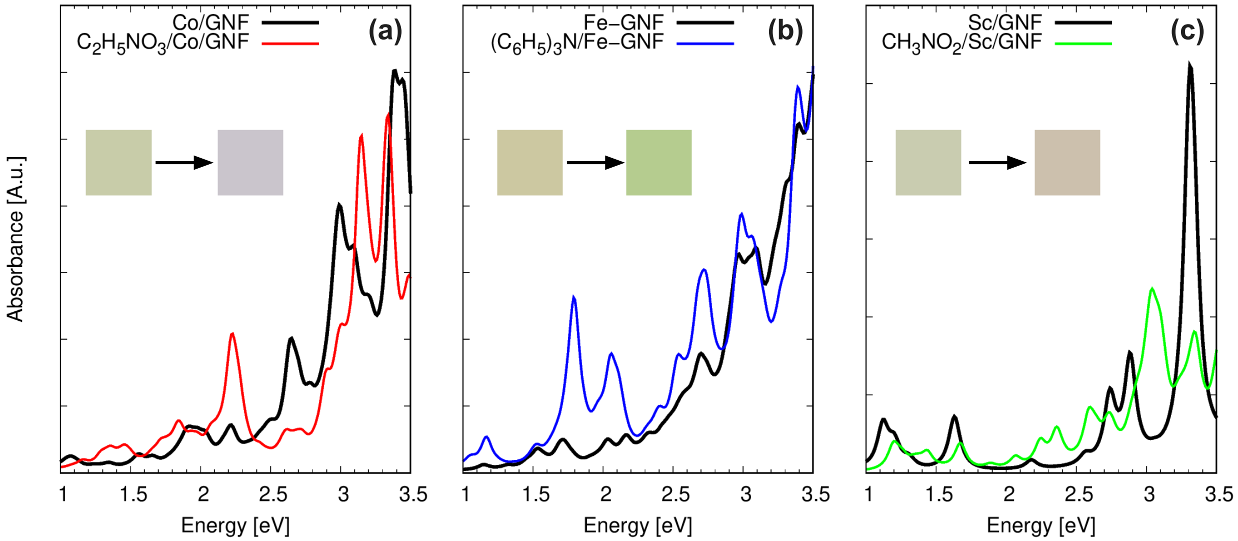

Figure S5: Comparison of the optical absorption spectra for some TM–functionalized GNF system in the absence (black) and presence (colored) of the adsorbed molecule, along with the expected color shifts shown in the insets representations. (a) Co–GNF and $C_2H_5NO_3$/Co–GNF. (b) Fe–GNF and $C_6H_5I_3N$/Fe–GNF. (c) Sc–GNF and CH_3NO_2/Sc–GNF.

Figure S5 summarizes additional examples of adsorption-induced optical variations for different TM–GNF systems. In all cases, molecule adsorption leads to spectral shifts and changes in peak intensities, which translate into distinct qualitative color responses, as indicated in the inset representations.

S5 Finite size effects

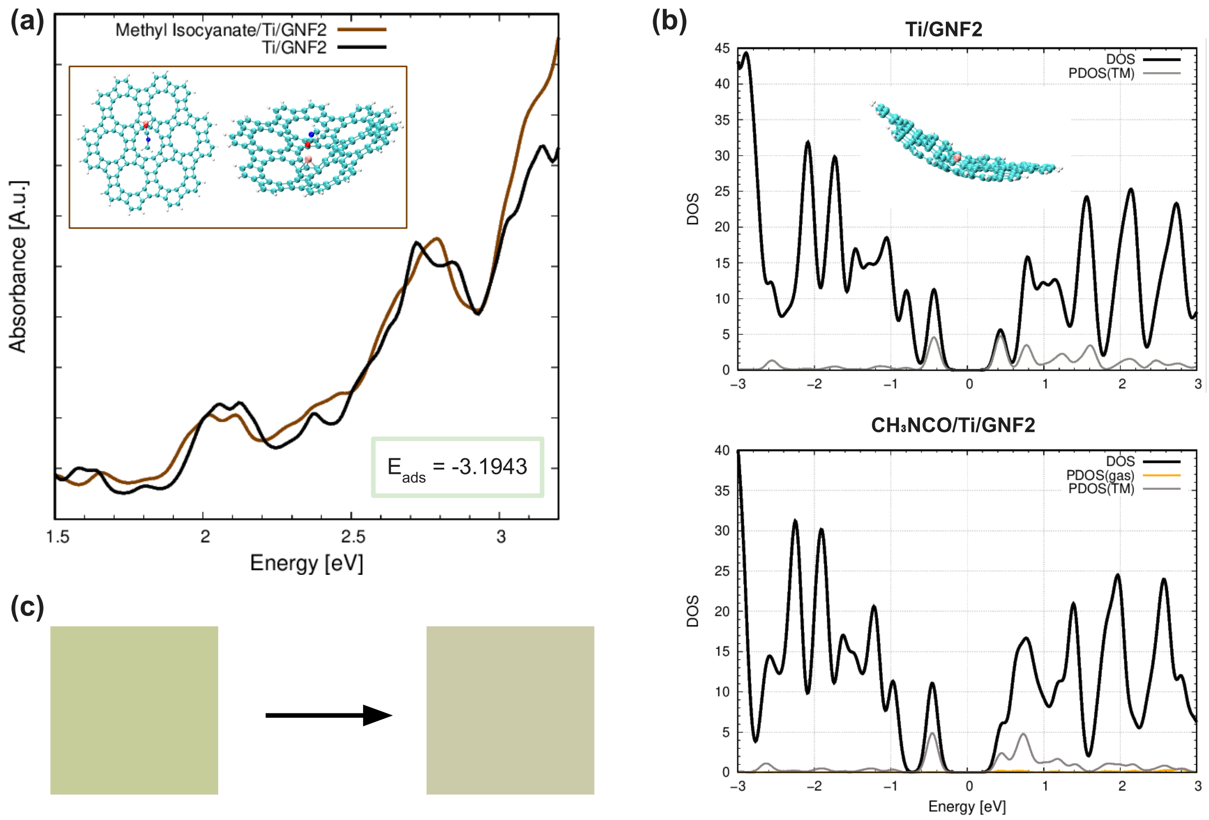

Figure S6: (a) Optical absorption spectra of Ti/GNF2 (black) and methyl-isocyanate/Ti/GNF2 (brown) systems. The inset is the optimized structure of the methyl-isocyanate/Ti/GNF2 complex. (b) Total and projected density of states (DOS and PDOS) for Ti/GNF2 (top) and methyl-isocyanate/Ti/GNF2 (bottom). The inset structure in the top is the optimized Ti/GNF2 complex. (c) Representation of the color change associated with the adsorption process.

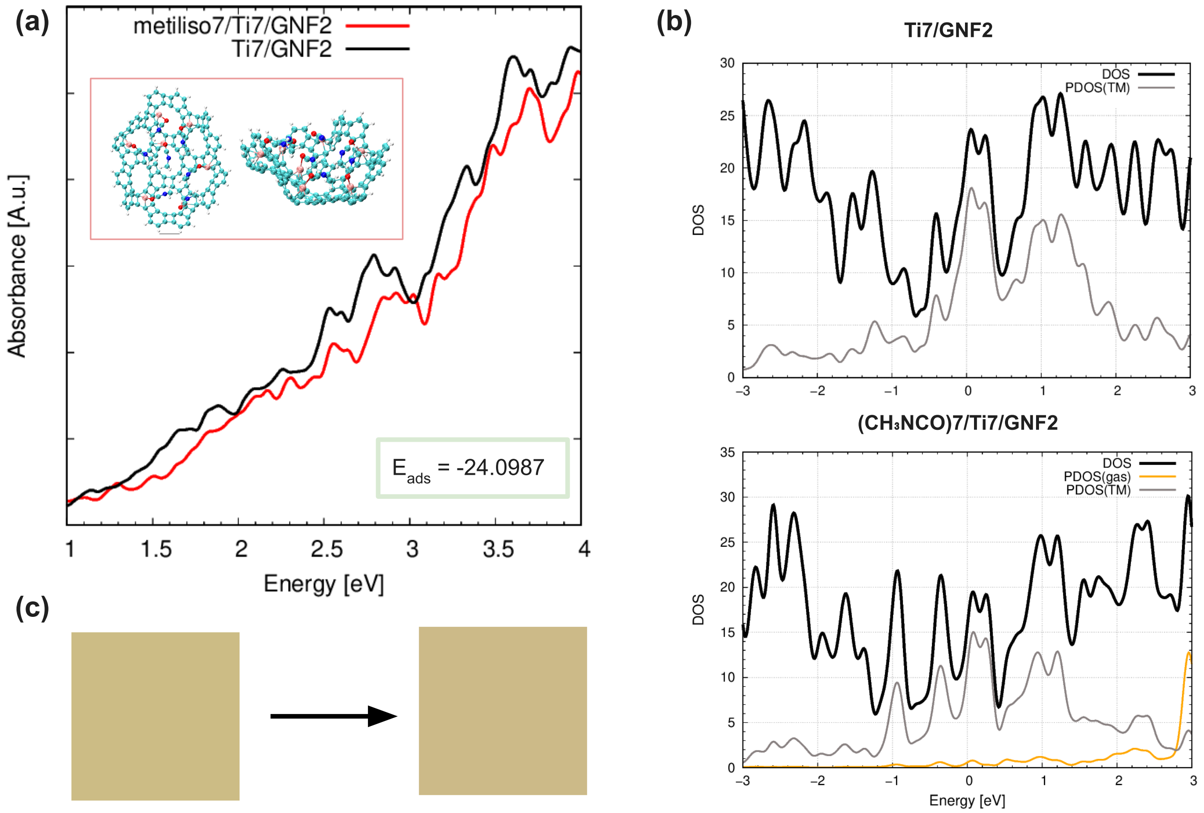

Figure S7: (a) Optical absorption spectra of Ti_7/GNF2 (black) and (methyl−isocyanate)$_7$/Ti_7/GNF2 (red) systems. The inset is the optimized structure of the gas/TM/GNF2 complex. (b) Total and projected density of states (DOS and PDOS) for the complex with (bottom) and without (top) multiple gas absorption. (c) Representation of the color change associated with the adsorption process.

To investigate whether the color changes observed in small graphenylene nanoflakes persist in larger systems, we extended our analysis to a larger hexagonal nanoflake ($C_{144}H_{24}$), denoted as GNF2. Such tests are, however, limited by computational cost, since TD-DFTB calculations for large systems with spin polarization become significantly more demanding. In this context, the methyl isocyanate/Ti combination was selected as a representative case. Although this system does not exhibit the strongest color shift or the most pronounced change in the absorption spectrum, it shows zero spin polarization, making TD-DFTB calculations easier.

For the adsorption of 1 Ti + 1 gas molecule on GNF2, the resulting absorption spectrum displays only minor changes relative to the Ti/GNF2 reference, with small shifts in peak

positions and intensities (Fig S6 (a)). Nevertheless, the DOS is modified upon adsorption, since the molecule placed near the transition-metal atom shifts molecular states toward the Fermi level (Fig S6 (b)).

Unlike the smaller flake, however, this modification in the DOS does not produce a substantial change in the absorption spectrum. This can arise from the large number of closely spaced states near the Fermi level - often degenerate - which increases the density of available excited states in the visible range. As a consequence, the system can become less sensitive to small local perturbations such as gas adsorption.

To amplify such perturbations, we scaled the system to multiple adsorption sites (7 Ti + 7 gas molecules). Multiple adsorption produced significant changes in the DOS, as seen in Fig. S7(b). However, these changes were again not reflected in the absorption spectrum, which preserved essentially the same qualitative behavior with and without the gas molecules (Fig S7 (a)). Thus, the predicted color variations remain negligible (Fig S7 (c)).

These observations can indicate that the pronounced color changes reported for smaller flakes arise primarily from the discrete nature of low-energy excitations in small GNF. In other words, excited states in the smaller flake are more energetically separated, so the gas-derived orbital has a much stronger effect on a given excitation.

References

(1) Aprà, E. et al. NWChem: Past, present, and future. *The Journal of Chemical Physics* **2020**, *152*.

(2) Perdew, J. P.; Burke, K.; Ernzerhof, M. Generalized Gradient Approximation Made Simple. *Physical Review Letters* **1996**, *77*, 3865–3868.

(3) Perdew, J. P.; Burke, K.; Ernzerhof, M. Generalized Gradient Approximation Made Simple [Phys. Rev. Lett. 77, 3865 (1996)]. *Physical Review Letters* **1997**, *78*, 1396–1396.

(4) Hellweg, A.; Rappoport, D. Development of new auxiliary basis functions of the Karlsruhe segmented contracted basis sets including diffuse basis functions (def2-SVPD, def2-TZVPPD, and def2-QVPPD) for RI-MP2 and RI-CC calculations. *Physical Chemistry Chemical Physics* **2015**, *17*, 1010–1017.

(5) Hourahine, B.; Sanna, S.; Aradi, B.; Köhler, C.; Niehaus, T.; Frauenheim, T. Self-Interaction and Strong Correlation in DFTB. *The Journal of Physical Chemistry A* **2007**, *111*, 5671–5677.
